# Transmuscular quadratus lumborum block versus thoracic paravertebral block for acute pain and quality of recovery after laparoscopic renal surgery: study protocol for a randomized controlled trial

**DOI:** 10.1186/s13063-019-3359-7

**Published:** 2019-05-20

**Authors:** Qing Yuan, Xulei Cui, Yuda Fei, Zhonghuang Xu, Yuguang Huang

**Affiliations:** 1 Department of Anesthesiology, Peking Union Medical College Hospital, Dongcheng-qu, Beijing, China; 2 Department of Anesthesiology, China Academy of Chinese Medical Sciences Eye Hospital, Shijingshan-qu, Beijing, China

**Keywords:** Quadratus lumborum block, Thoracic paravertebral block, Laparoscopic surgery

## Abstract

**Background:**

Quadratus lumborum block (QLB) is increasingly gaining popularity as a novel abdominal truncal block in abdominal surgery; however, the mechanism of QLB is not yet thoroughly illustrated. The focus of our study is transmuscular QLB (TMQLB), as the latest anatomical evidence shows that anesthetics spread into the thoracic paravertebral space to exert an analgesic effect. Therefore, we designed this study to compare TMQLB with thoracic paravertebral block (TPVB) in laparoscopic renal surgery in the hope of providing clinical evidence on the analgesic mechanism of TMQLB and its application in laparoscopic renal surgery.

**Methods:**

This trial is a prospective, randomized, single-center, open-label, parallel, three-arm, non-inferiority trial. We intend to include 120 participants undergoing laparoscopic nephrectomy and before surgery they will be randomized into three groups for postoperative pain control: TMQLB experimental group 1 (0.4 ml/kg body weight 0.5% ropivacaine), TMQLB experimental group 2 (0.6 ml/kg body weight 0.5% ropivacaine) or TPVB control group (0.4 ml/kg body weight 0.5% ropivacaine at vertebra T10). Patients will be excluded if they have allergy to anesthetics, infection at the injection site, are on coagulopathy or anticoagulants, on analgesics for chronic illness, have history of substance abuse or have a barrier to communication. Morphine is given in boluses of 1.5~2 mg by intravenous patient-controlled analgesia (IPCA) in the first 48 h after surgery. The primary outcome is the difference between TMQLB group 1 and the TPVB group in the mean visual analogue scale (VAS) pain score in the first 24 h after surgery. Secondary outcomes are the difference between TMQLB group 2 and the TPVB group in the mean VAS score in the first 24 h after surgery, cumulative morphine consumption, long-term pain control, dermatomal distribution of sensory loss, nausea score, pruritus score, ambulation time, time till recovery of bowel movement, quality of recovery, postoperative length of hospital stay and patient satisfaction with anesthesia. Safety data on procedure-related complications will also be summarized.

**Discussion:**

This will the first randomized controlled trial to compare TMQLB with TPVB for analgesia in laparoscopic surgery. This trial aims to provide important clinical evidence to elaborate on the analgesic mechanism of TMQLB.

**Trial registration:**

ClinicalTrials.gov, NCT03414281. Registered on 9 January 2018.

**Electronic supplementary material:**

The online version of this article (10.1186/s13063-019-3359-7) contains supplementary material, which is available to authorized users.

## Strengths and limitations of this study

- This will the first randomized controlled trial to compare transmuscular quadratus lumborum block with thoracic paravertebral block for analgesia in laparoscopic surgery
- This trial will provide important clinical evidence to elaborate the analgesic mechanism of transmuscular quadratus lumborum block
- The methodology of this trial has strengths that include a well-designed intervention, computerized randomization, blinded assessment and data-analysis and appropriate estimation of the sample size
- The relatively small sample size may not be adequate to detect a difference in safety difference between the two block techniques

## Background

Quadratus lumborum block (QLB) is a novel abdominal truncal block providing analgesia for abdominal surgery including cesarean section, laparoscopy, colostomy, pyeloplasty and hernia repair [1–5]. Currently, there are four different approaches for QLB, with local anesthetic injected around the quadratus lumborum (QL) muscle from various directions. The QLB 1 was first proposed by Blanco in 2007: the needle is inserted into the plane between the psoas major muscle and the QL muscle and the local anesthetic is injected into the anterolateral margin of the QL muscle, which is also known as the lateral QLB [6]. The QLB 2 approach involves injection of the anesthetic posterolateral to the QL muscle [7], and so QLB 2 is also referred to as posterior QLB. Later, Børglum et al. [8] described another approach, transmuscular QLB (TMQLB), whereby the needle is advanced through the latissimus dorsi and the QL muscle in a posterior-anterior direction with the injection performed anterior to the QL muscle. This approach is also referred to as QLB 3 or anterior QLB. Last but not least, there is intramuscular QLB, whereby the anesthetic is injected directly into the QL muscle [5].

When first proposed by Børglum, TMQLB was designed to alleviate pain in intraperitoneal and retroperitoneal surgical procedures. The original hypothesis of the analgesic mechanism of TMQLB was that the injectate deposited within the plane between the QL and psoas major muscle would spread cranially to the thoracic paravertebral space to exert its analgesic effect. But it is not until recently that solid anatomical findings have verified this assumption. Børglum et al. [9] conducted a cadaver study investigating the spread of dye solution after TMQLB and showed convincingly that the injectate could reach the thoracic sympathetic trunk and the ventral rami of the lower thoracic (T9–T12) spinal nerves in the thoracic paravertebral space. The injectate spread cranially from the point of lumbar administration between the QL muscle and the psoas major muscle to reach the paravertebral space via a pathway posterior to the medial and lateral arcuate ligaments.

Based on this anatomical evidence, we could assume that the analgesic effect of TMQLB will be comparable to thoracic paravertebral block (TPVB) because both approaches exert analgesic effects by infiltrating the thoracic somatic and sympathetic nerves in the thoracic paravertebral space. Despite infrequent complications with TPVB, concerns remain about the possibility of pneumothorax, vascular or dural puncture and epidural or intrathecal spread. Moreover, as a deep block performed in a non-compressible space, TPVB involves a risk of bleeding in the thoracic paravertebral space, which is difficult to deal with and clotting is dependent on the patient’s hemostasis [10]. TPVB is thus relatively contraindicated in patients on anticoagulation medication as suggested by the anticoagulation guidelines of American Society of Regional Anesthesia and Pain Medicine (ASRA) [18]. By contrast, the risk of injury associated with TMQLB is lower because the passage of the needle and the endpoint is within muscle and the fascial plane, distant from the peritoneal cavity, abdominal organs, large blood vessels and nerves. Henceforth, if clinical evidence demonstrates that TMQLB provides similar analgesic effect to TPVB, the complications associated with TPVB could be avoided with the application of TMQLB. However, it is regrettable that high-quality randomized clinical trials (RCTs) on the efficacy and safety of TMQLB in abdominal and retroperitoneal surgery are scarce and there has been no direct comparison between TMQLB and TPVB in these scenarios.

### Objectives

The aim of this study is to design a prospective RCT to compare TMQLB with TPVB in terms of the efficacy of analgesia and the quality of recovery in laparoscopic renal surgery. Our hypothesis is the analgesic efficacy of TMQLB in laparoscopic renal surgery is non-inferior to that of TPVB.

## Methods

### Study design

**Fig. 1:**
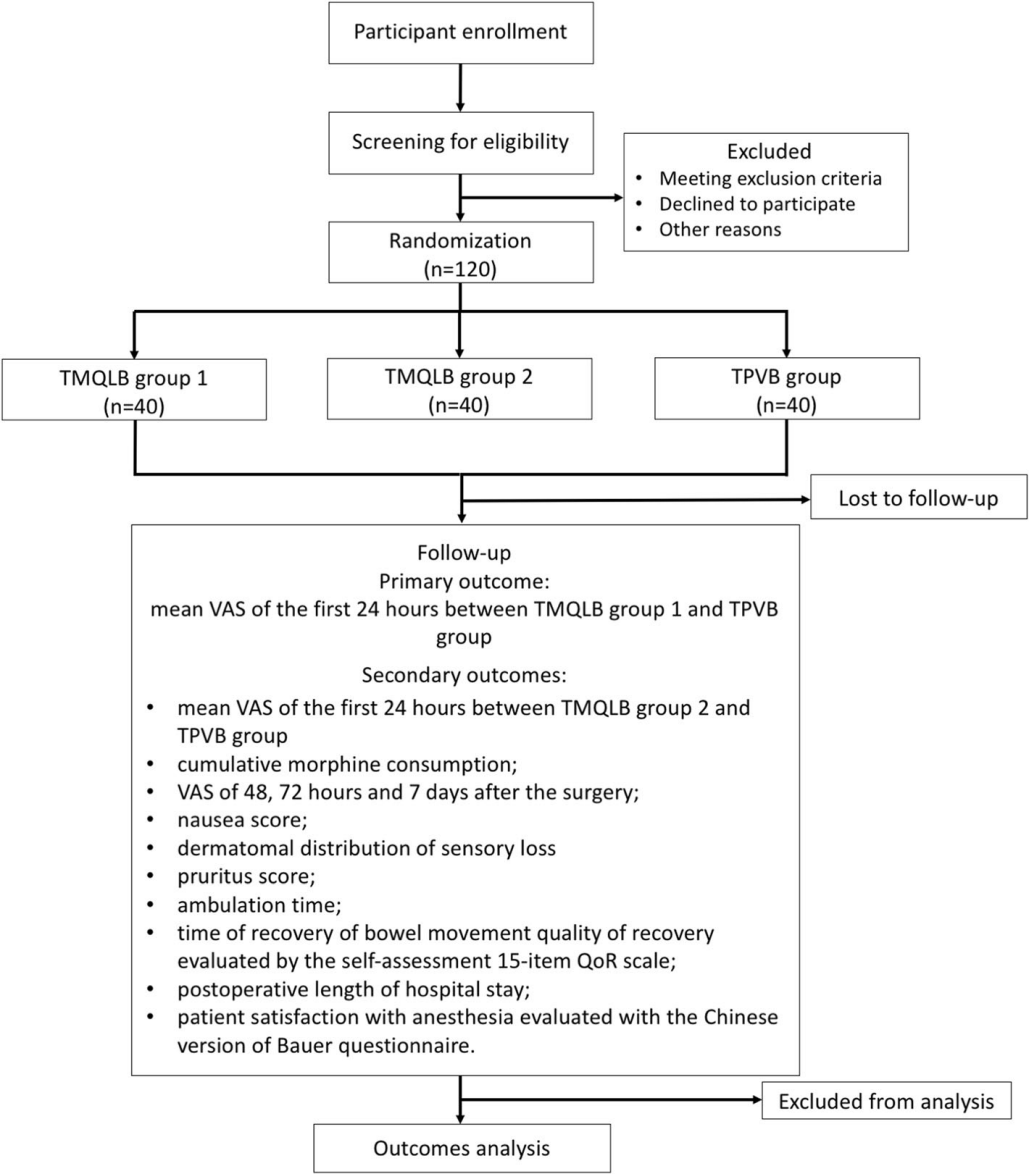
Flow diagram of the study. TMQLB, transmuscular quadratus lumborum block; TPVB, thoracic paravertebral block; VAS, visual analogue score; QoR, quality of recovery

Our trial is a prospective, randomized, single-center, open-label, parallel, 3-arm, non-inferiority trial, the objective of which is to evaluate pain relief and the quality of recovery in laparoscopic renal surgery using TMQLB compared with TPVB. The overall trial scheme is illustrated in Fig. 1 (see Additional file 1 for the SPIRIT Checklist). Institutional research ethics board approval was obtained from Peking Union Medical College Hospital (PUMCH). The trial will be conducted at PUMCH in accordance with the International Conference on Harmonisation (ICH) guideline for Good Clinical Practice (GCP). Full written informed consent will be obtained from each participant by a qualified member of the research team before the intervention. This trial was registered with the US National Institutes of Health Clinical Trials registry (NCT03414281). Participants will be randomized into three groups receiving TMQLB (two dosing groups) or TPVB for postoperative pain control.

### Eligibility criteria

#### Inclusion criteria

Participants enrolled in this trial will be:

- 17~80 years of age
- Categorized as American Society of Anesthesiologists (ASA) class I–III
- Undergoing laparoscopic nephrectomy

#### Exclusion criteria

Patients will be excluded if they:

- Have known allergy to the anesthetics being used
- Have infection at injection site
- Are receiving coagulopathy or have history of anticoagulant use
- Are taking analgesics for chronic illness or have history of substance abuse
- Are unable to appropriately describe to the investigators their postoperative pain or recovery (e.g., language barrier or neuropsychiatric disorder)

### Interventions

Intravenous access and standard ASA monitoring will be established after patients arrive at the preoperative holding area. Premedication with 1–2 mg midazolam will be considered depending on the patient’s condition. All patients will receive ultrasound-guided block administered by the same experienced attending anesthesiologist. After the block, the anesthesia induction regimen will be as follows: propofol (2 mg/kg body weight), fentanyl (1 μg/kg body weight), and rocuronium (0.9 mg/kg body weight). Patients will be intubated endotracheally. During the surgery, sevoflurane and a mixture of O_2_/N_2_O will be used for maintenance to keep the bispectral index (BIS) within the range of 40–60. Fentanyl will be administered as needed to control the heart rate and blood pressure to within ± 20% of the baseline values.

#### TMQLB experimental group 1

For participants in the TMQLB group 1, the QLB will be performed using the transmuscular approach. The patient is placed in the lateral position. The patient is scanned using the curved (C1–5) probe of the Philip CX50 Ultrasound Scanner is used and is located vertical to the iliac crest at the posterior axillary line to find the Shamrock sign. The 22-G needle is then inserted in plane and directed to the QL muscle. After the proper position of the needle tip between the psoas major muscle and the QL muscle is confirmed, ropivacaine 0.5% at 0. 4 ml/kg body weight with adrenaline at a ratio of 1:200,000 is injected into the interfascial plane. The effective block is confirmed by the sign of local anesthetic spreading around the QL muscle and the loss of sensory function in the area. If there is no block of sensory function after 45–60 min, then it is defined as an unsuccessful block. All interventions will be performed by the same experienced attending anesthesiologist.

#### TMQLB experimental group 2

The intervention in TMQLB group 2 conforms to that used in TMQLB group 1 except that the dose of ropivacaine is 0. 6 ml/kg body weight.

#### TPVB control group

In the TPVB group, the patient is placed in the lateral position, the spinous processes of vertebra T10 are identified and marks are made 2 cm lateral to the spinous processes. The linear (L12–3) probe of the Philips CX50 is placed transversely at the mark to identify the paravertebral space. Then a 22-G needle is inserted in-plane in a lateral to medial direction and advanced until the tip reaches the paravertebral space surrounded by the parietal pleura and the superior costotransverse ligament. Then, ropivacaine 0.5% at 0.4 ml/kg body weight with adrenaline at a ratio of 1:200,000 is injected into the paravertebral space of vertebra T10. The block is confirmed as correct by the sign of the parietal pleura being pressed down by the local anesthetic. A successful block is defined as loss of sensory function in the area.

#### Postoperative analgesia

Each participant will receive standard postoperative intravenous patient-controlled analgesia (IPCA) at the end of surgery. Morphine is given in boluses of 1.5–2 mg as needed without baseline infusion in the first 48 h postoperatively, with a lockout time of 10 min. The 1-h limit is 6–8 mg morphine.

### Randomization and allocation concealment

All eligible patients will be randomized to TMQLB group 1, TMQLB group 2 or the TPVB group in a ratio of 1:1:1, using the computerized SPSS package (version 22; SPSS Inc., Chicago, IL, USA). The randomization sequence will be computer-generated by a professional statistician who is not involved in the implementation and statistical analysis of the study. Allocation concealment will be ensured by using sealed, opaque, sequentially numbered envelopes. These assignment envelopes will only be opened after the inclusion of the patient in the study. A study coordinator will be responsible for enrolling patients, obtaining informed consent and requesting randomization.

### Blinding

Because of the nature of the intervention, the anesthesiologist who performs the intervention and the trial participants cannot be blinded to the allocation of the intervention. Therefore, this is an open-label study whereby the investigator and patients are not blinded. However, the outcome assessor and statistician who performs the data analysis are blinded to the allocated intervention. The participant’s allocated intervention will not be revealed until the final data analysis is completed.

### Outcomes

#### Primary outcome

The primary outcome of this trial is mean visual analogue scale (VAS) score for pain in the first postoperative 24 h, between TMQLB group 1 and the TPVB group. The VAS score will be recorded at 0, 2, 4, 8, 12 and 24 h after surgery, and the primary outcome will be calculated as the mean VAS scores measured at these time points. The VAS is an internationally recognized scale for assessment of pain on an 11-point scale ranging from 0 to 10 points, with 0 defined as no pain and 10 defined as the worst pain imaginable.

#### Secondary outcomes

Secondary outcomes are as follows:

- Mean VAS score in the first postoperative 24 h between TMQLB group 2 and the TPVB group. In theory, the dose of local anesthetic in TMQLB group 1 is lower than that in TMQLB group 2, hence, if group 1 satisfies the non-inferiority hypothesis, the mean VAS score in group 2 should also be non-inferior to that in the TPVB group. If not, the results of the comparison between TMQLB group 2 and the control will be hypothesis-generating.
- Cumulative morphine consumption, which will be registered at 0, 2, 4, 8, 12, 24, 48 and at 72 h and 7 days after surgery, and will be calculated as the sum of the values.
- Long-term pain control, which will be evaluated by VAS at 48 and 72 h and at 7 days after surgery.
- Dermatomal distribution of sensory loss, which will be evaluated at 10, 20, 30 and 40 min after the intervention by pinprick test using Von Frey filaments.
- Nausea score, which will be recorded at 0, 2, 4, 8, 12, 24 and 48 h after surgery.
- Pruritus score, which will be recorded at 0, 2, 4, 8, 12, 24 and 48 h after surgery.
- Ambulation time
- Time of recovery of bowel movement (defined as the time to first flatus).
- Quality of recovery evaluated by the self-assessment quality of recovery (QoR) scale at 3 days and 5 days after surgery. The QoR is a 15-item questionnaire, scored on a scale of 0–10 pertaining to the patient’s comfort, support system, pain, wellbeing and ability to carry out daily activities, where 0 indicates none of the time and 10 indicates all of the time [11].
- Length of postoperative of hospital stay.
- Patient satisfaction with anesthesia evaluated using the Chinese version of the Bauer questionnaire at 48 h after surgery [12].

### Trial safety

The ultrasound-guided block will be performed only after intravenous access and standard monitoring is established for the patient. The intervention will be stopped if any adverse event occurs during the procedure. After the intervention, research personnel will continue monitoring the patient to detect any possible adverse events until the patient enters the operating room. Monitoring will continue in the postanesthesia care unit (PACU) and the surgical ward. Research personnel will follow up the patient at 0, 2, 4, 8, 12, 24, 48 and 72 h and at 7 days after surgery. All adverse events recorded in this period will be reported to the adverse event registration system of the hospital.

### Data collection and management

Baseline data will be collected, including age, gender, weight, body mass index (BMI), ASA grading, duration of surgery and vital signs. We will also document intervention-related complications including hematoma, organ injuries, and lower extremity weakness in TMQLB, and vascular puncture, pleural puncture, pneumothorax and dural puncture in TPVB. All personal information will be registered in an environment limited to medical personnel to maintain absolute confidentiality.

A web-based electronic data capture (EDC) system will be built for data collection. During the follow-up period, the outcome assessor will record the data on standardized paper case report forms (CRFs) to ensure all data points are noted. After the paper CRF is completed for each patient, it will be entered into the electronic CRF and saved in the trial database. The database is protected by password, but still allows for data audits to check completeness and accuracy. Data lockup will be implemented on completion of the study and the researchers will not be able to modify the data. The allocation will be blinded until all data analyses are completed. The paper CRFs and the electronic database will be preserved for at least 5 years after publication in case of any inquiry.

### Sample size calculation

The primary outcome of this non-inferiority trial is the mean VAS score in the first postoperative 24 h in TMQLB group 1 (ropavacaine 0.4 ml/kg body weight) and the TPVB group. Hence, we used the sample size estimation with the non-inferiority test for the difference between two means using PASS 15 software. A higher VAS score in clinical practice indicates that the patients suffer worse pain. No non-parametric adjustment was used in the sample size calculation. We used statistical power of 90% and two-sided α of 0.05. Based on our pilot study, the VAS scores in TMQLB group 1 and the TPVB group were 3.35 and 3.08, respectively, and the standard deviation (SD) of the mean VAS score was 0.5. The non-inferiority margin was set as 0.616 (20% of the VAS score in the TPVB group). The target sample size for each group is at least 36 participants. Considering a potential dropout rate of 10% in the postoperative data collection, we plan to include 40 patients in each group.

### Data analysis

We will first describe the baseline characteristics of the participants and compare the group difference using the standardized difference; a standardized difference smaller than 0.2 will be considered acceptable deviation between groups [19]. The distribution of the variables will be checked by visual inspection of the histogram. Normally and non-normally distributed variables will be described by the mean ± SD or median (interquartile range (IQR)).

In all data analyses, we will first compare TMQLB group 1 with the TPVB group. If the result is negative, then we will compare data for TMQLB group 2 with the TPVB group to explore whether increasing the amount of local anesthetic would improve the outcome of TMQLB.

Since the primary outcome of the mean VAS score in the first postoperative 24 h is generally normally distributed as reported by Dai et al. [13], the result will be expressed as mean ± SD. Student’s *t* test will be used to compare the difference in the mean postoperative VAS score between TMQLB group 1 and the TPVB group, and the mean difference with corresponding one-sided 95% CI will be calculated. If the upper bound of the one-sided 95% CI is smaller than 0.616, we will reject the null hypothesis in the non-inferiority test and conclude that the mean VAS score in the first postoperative 24 h in TMQLB group 1 is non-inferior to that in the TPVB group.

For the secondary outcomes, the mean difference in the VAS score between TMQLB group 2 and TPVB group, cumulative morphine consumption and long-term VAS scores will be analyzed using the non-inferiority test, while other variables will be analyzed using the superiority test. For continuous data, Student’s *t* test will be used for parametric data analysis and the Mann-Whitney test will be used for non-parametric data analysis, including data on morphine consumption which are often reported as highly skewed. The time-to-event data including time of bowel movement recovery, ambulation time and length of hospital stay will be analyzed by Kaplan-Meier estimates to obtain the survival curves and then the log-rank test will be used to compare curves between groups.

In the case of data missing or dropouts, data analysis will be performed according to the intention-to-treat principle. The results of this trial will be reported according to the Consolidated Standards of Reporting Trials (CONSORT) statement [17]. All data analysis will be performed using SPSS software (version 22; SPSS Inc., Chicago, IL, USA).

## Discussion

Although QLB is gaining popularity as analgesia in abdominal surgery, there has been limited clinical study of its clinical efficacy compared with other truncal block techniques. Most of the current studies concentrate on the comparison of QLB with other abdominal blocks, especially the transversus abdominis plane (TAP) block, because QLB was originally proposed as a different form of TAP [4, 5, 15, 16]. Also, the analgesic mechanism of TMQLB is still controversial. The anatomical work performed by Børglum et al. [9] reveals that in TMQLB the injectate spreads cephalad to the thoracic paravertebral space. This convincing study prompted us to speculate that TMQLB may have a similar analgesic profile to TPVB, which is essentially a thoracic truncal block, and this inspired us to conduct a trial to compare these two block techniques and further validate the theory from a clinical aspect.

The expected results will provide clinical evidence to verify the analgesic mechanism of TMQLB put forward by Børglum and promote its application in intraperitoneal and retroperitoneal surgery. Furthermore, if the efficiency of TMQLB is equal to that of TPVB, TMQLB could replace TPVB in abdominal surgery because it is relatively safe and convenient to perform, while TPVB implicates a possibility of pneumothorax, dural puncture and hematoma in the thoracic paravertebral space [10, 14].

As the mechanism of the injectate of TMQLB reaching the paravertebral space is primarily by spreading via the pathway posterior to the arcuate ligaments, it could be assumed that the amount of the injectate will consequently determine the extent to which the thoracic nerves are blocked. Henceforth, we designed three intervention groups with two dosing groups for TMQLB to explore whether the amount of injectate would improve the coverage and analgesic effect. This may also provide a clue to the appropriate dosage for anesthetics in TMQLB.

To the best of our knowledge, the present study will be the first to compare TMQLB and TPVB for the purpose of assessing the clinical effectiveness and safety of TMQLB in laparoscopic renal surgery. The proposed trial is a prospective, randomized and blinded-analysis trial with rigorous methodology to avoid potential risk of bias. Since the nature of the intervention makes it impossible for the participant and the investigator performing the block to be blinded, the outcome assessor and statistician will be blinded to the allocation to keep the data analysis unbiased. In addition, the strict process of randomization and allocation of each participant could reduce the risk of selection bias.

If our trial yields positive results, there is potential that TMQLB could be recommended as an alternative nerve block for postoperative analgesia in abdominal surgery, to circumvent the need for TPVB.

### Trial status

This trial was initiated in January 2019. At the time of manuscript submission, the recruitment is ongoing.

## Additional file

Additional file 1: SPIRIT 2013 checklist: Recommended items to address in a clinical trial protocol and related documents*. (DOC 120 kb)

## Abbreviations

ASA: American Society of Anesthesiologists
ASRA: American Society of Regional Anesthesia and Pain Medicine
BMI: Body mass index
CRF: Case report form
EDC: Electronic data capture
GCP: Good Clinical Practice
ICH: International Conference on Harmonisation
IPCA: Intravenous patient-controlled analgesia
IQR: Interquartile range
PACU: Postanesthesia care unit
PUMCH: Peking Union Medical College Hospital
QL: Quadratus lumborum
QLB: Quadratus lumborum block
QoR: Quality of recovery
RCT: Randomized clinical trial
SD: Standard deviation
TMQLB: Transmuscular quadratus lumborum block
TPVB: Thoracic paravertebral block
VAS: Visual analogue scale
